# A novel tool for phase contrast MR-derived pulse wave velocity measurement - validation against applanation tonometry and phantom studies

**DOI:** 10.1186/1532-429X-17-S1-P40

**Published:** 2015-02-03

**Authors:** Karolina Dorniak, Marcin Hellmann, Dorota Rawicz-Zegrzda, Maria Wesierska, Agnieszka Sabisz, Edyta Szurowska, Maria Dudziak, Einar Heiberg

**Affiliations:** 1 Department of Noninvasive Cardiac Diagnostics, Medical University of Gdansk, Gdansk, Poland; 2 2nd Department of Radiology, Medical University of Gdansk, Gdansk, Poland; 3 Department of Clinical Physiology, Lund University, Lund, Sweden; 4 Medviso AB, Lund, Sweden

## Background

Arterial stiffness is one of the most potent prognostic factors of cardiovascular morbidity and mortality. Its surrogate parameter, pulse wave velocity (PWV), is most commonly assessed by carotid-femoral applanation tonometry (AT). Limited availability of the AT equipment limits its application in clinical practice. Phase contrast cardiac magnetic resonance (CMR) offers insight in arterial stiffness at no extra cost, without significant protocol extension. As CMR accessibility increases, validated post-processing tools for CMR-derived PWV measurement are needed.

The aim of the study was to provide a validated, freely available tool to measure PWV using a routine CMR protocol.

## Methods

AT- and CMR-derived PWV measurements were compared in 21 subjects (10 healthy subjects aged 28±8 (16-44) yrs in whom cardiovascular disease was excluded based on clinical assessment and CMR result and 11 patients with hyperlipidemia and/or hypertension aged 57,8 ± 7,5 yrs).

AT-derived PWV measurements were done by the carotid [C] and femoral [F] applanation pulse wave recording and body surface approximation of the distance travelled (suprasternal notch to [F] - suprasternal notch to [C]), using SphygmoCor, (AtCor Medical, Australia). Phase contrast CMR images were acquired with a 3T or a 1,5T scanner (Achieva 3T TX, Philips, or Aera 1,5T, Siemens), and PWV was assessed, based on ascending and thoracic aortic flow data and direct aortic length measurements. In addition to scanning patients and healthy subjects, computer phantoms were constructed based on true aortic curves and known time delays. A PWV quantification method based on curve foot transit time was implemented in the software Segment (http://segment.heiberg.se).

## Results

**Figure 1:**
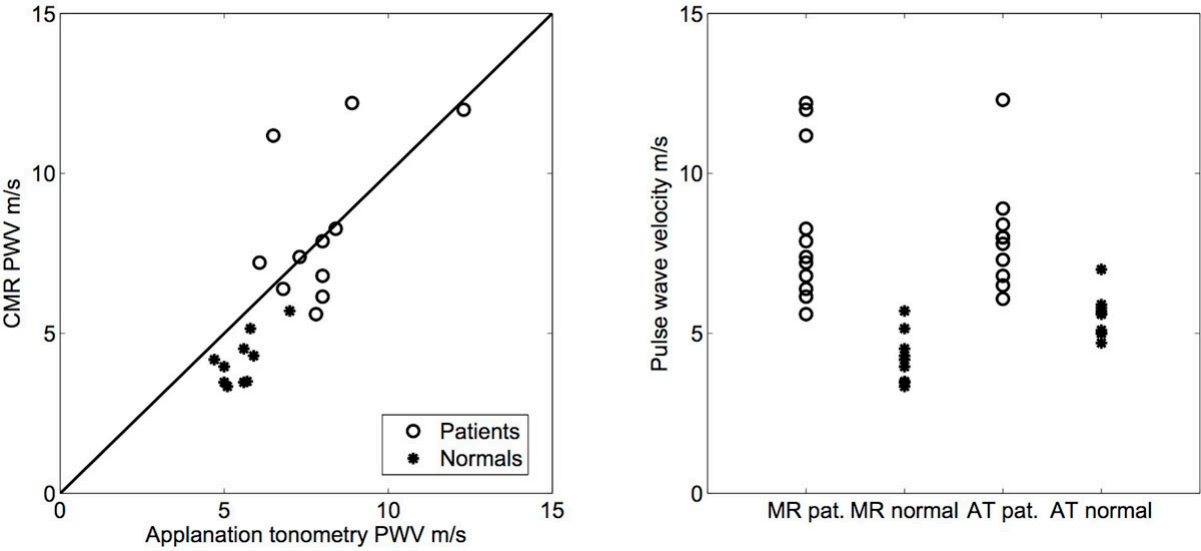
AT-PWV and CMR-PWV correlation plot (left, R=0,77) and PWV value distribution in the two subgroups by both methods.

**Figure 2:**
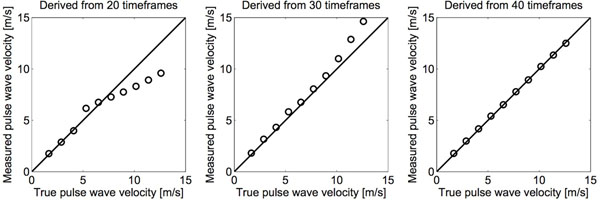
Results from phantom experiments with different number of time frames in the measured phase contrast images.

Mean AT-PWV values were 5.54±0.65 m/s and 8.0±1.65 m/s and mean CMR-PWV were 4.15±0.79 m/s and 8.19±2.60 m/s in healthy subjects and in patients, respectively. CMR-PWV agreed well with AT-PWV measurements (Figure 1, left panel, R=0,77). For CMR-PWV there were overlap with one patients compared to normals, whereas there was an overlap with three patients for AT-PWV (Figure 1, right panel). Intraobserver variability of CMR-PWV was -0.03±0.57 m/s, and inter observer variability was 0.09±0.49 m/s. From the phantom experiments it was found that in order to accurately quantify higher PWVs, a time resolution of at least 40 timeframes is required (Figure 2).

## Conclusions

A freely available tool for CMR-derived PWV measurement was successfully validated against applanation tonometry as well as in phantom studies. These results indicate that aortic PWV measurements incorporated in a routine CMR examination correlate well with carotid-femoral AT-PWV measurements in individuals without detectable cardiovascular disease and in patients. CMR-derived PWV analysis has low intraobserver and interobserver variability, both in healthy subjects and in patients.

## Funding

N/A.

